# Fungus–Fungus Association of *Boletus griseus* and *Hypomyces chrysospermus* and Cadmium Resistance Characteristics of Symbiotic Fungus *Hypomyces chrysospermus*

**DOI:** 10.3390/jof8060578

**Published:** 2022-05-27

**Authors:** Zhen Tian, Yunan Wang, Yongliang Zhuang, Chunze Mao, Yujia Shi, Liping Sun

**Affiliations:** Faculty of Food Science and Engineering, Kunming University of Science and Technology, No. 727, Jingming South Road, Chenggong District, Kunming 650500, China; tianzhen2020@yeah.net (Z.T.); wyn9708@163.com (Y.W.); ylzhuang@kmust.edu.cn (Y.Z.); kmczmao@163.com (C.M.); kmyjshi@163.com (Y.S.)

**Keywords:** *Boletus griseus*, *Hypomyces chrysospermus*, cadmium, symbiotic association

## Abstract

Fungi bioaccumulation of heavy metals is a promising approach to remediate polluted soil and water. *Boletus griseus* could accumulate high amounts of Cd, even in a natural habitat with low Cd contents. This study found a symbiotic association of *B. griseus* with a fungus. The symbiotic fungus was isolated and identified as *Hypomyces chrysospermus*. The isolated strain had a strong ability to tolerate Cd. The minimum inhibitory concentration of Cd of fungal growth was 200 mg·L^−^^1^. The Cd bioaccumulation capacity of the fungus reached 10.03 mg·g^−1^. The biomass production of the fungus was promoted by 20 mg·L^−1^ Cd. However, high concentrations of Cd suppressed fungal growth and significantly altered the morphology and fine texture of fungal hyphae and chlamydospores. The immobilization effects of the cell wall and acid compounds and antioxidant enzymes were employed by the fungus to alleviate the toxic effects of Cd. The results not only demonstrate a new insight into the Cd bioconcentration mechanisms of *B. griseus* but also provide a potential bioremediation fungus for Cd contamination.

## 1. Introduction

*Boletus griseus* is a common wild-grown edible mushroom in Yunnan Province, and it is a member of the genus *Boletus* in the family *Boletaceae* [1]. Studies have confirmed that *B. griseus* can accumulate high amounts of Cd in fruiting bodies, even from natural habitats with low Cd contents in the soil matrix [2,3,4]. The Cd contents of 153 samples of *B. griseus* were found to range from 1.61–42.67 mg·kg^−1^ while those in soil ranged from 0.03–0.57 mg·kg^−1^. The bioconcentration factors of *B. griseus* for Cd were 24–386 [4]. The factors affecting Cd migration from soil to *B. griseus* were investigated. Principal component analysis elucidated that the soil physical-chemical properties, such as the Cd content in soil, electrical conductivity, total carbon, total nitrogen, pH, and dissolved organic carbon, were factors that affected the Cd accumulation of *B. griseus* [4]. Macrofungi are known to effectively accumulate higher concentrations of metals and metalloids than vascular plants [5]. Therefore, the accumulation of elements in mushrooms has attracted significant research attention [6,7,8]. Considering the natural values of Cd in mushrooms, *B. griseus* showed significantly higher Cd contents than most reported mushrooms, except for the family Agaricaceae [9].

**Figure 1:**
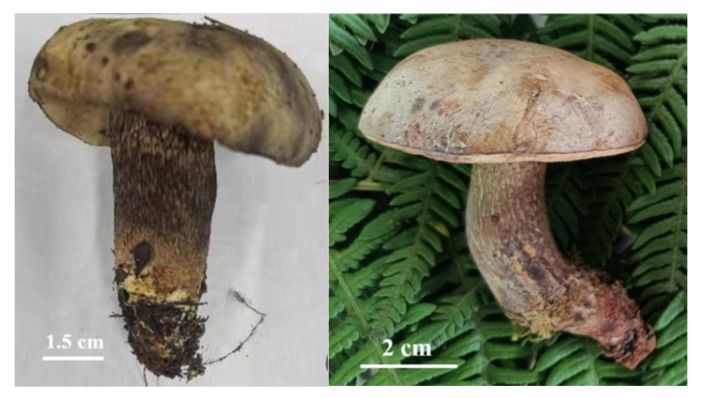
Normal-developed sporocarps of *B. griseus*.

**Figure 2:**
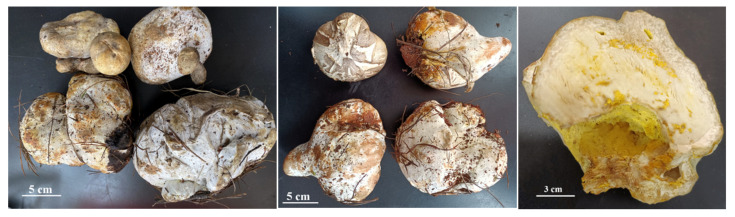
The symbiotic fruiting bodies of *B. griseus*–mycoparasitic fungus and their vertical section.

*B. griseus* is a kind of ectomycorrhizal mushroom, and is symbiotically associated with a large number of trees and shrubs. It can be found along the forest edge of pine-oak mixed forests in Yunnan Province [10]. When sampling, two phenotypes of the sporocarps of *B. griseus* were found, namely, normal-developed sporocarps (Figure 1) and symbiotic fruiting bodies of *B. griseus*–mycoparasitic fungus (Figure 2). Owing to the geographical location and ecological, climatic, topographic, and geological factors, Yunnan Province is rich in fungal biodiversity, with more than 882 edible species and an average annual yield of 500,000 tons. Many studies have focused on the resource survey, annual yield, bioactivities and bioactive components, and food safety of wild edible mushrooms [2,11,12,13,14,15,16]. However, few reports have studied the fungicolous fungi and host–parasite relationships in wild edible mushrooms, except for the genus *Hypomyces* [17,18].

*Hypomyces* is a genus in the family *Hypocreaceae* with more than 150 species, including the most characteristic mycoparasites of diverse fungal hosts of agarics, boletes, russules, thelephores, and polypores [17]. Most species of *Hypomyces* are obligatory parasites, growing only on a specific host. The parasites of *Hypomyces* usually cause systematic infection and mummification of the host fruiting bodies [18]. Douhan and Rizzo collected 22 isolates from 21 infected bolete and one infected agaric in California oak woodlands, among which 20 isolates from individual bolete hosts were identified as *H. microspermus* and *H. chrysospermus* [17]. Kaygusuz et al. described *H. chrysospermus* isolated from infected *Suillus luteus* as a new record in Turkey [18]. Thus, the genus *Hypomyces* exclusively comprises boleticolous species.

**Figure 3:**
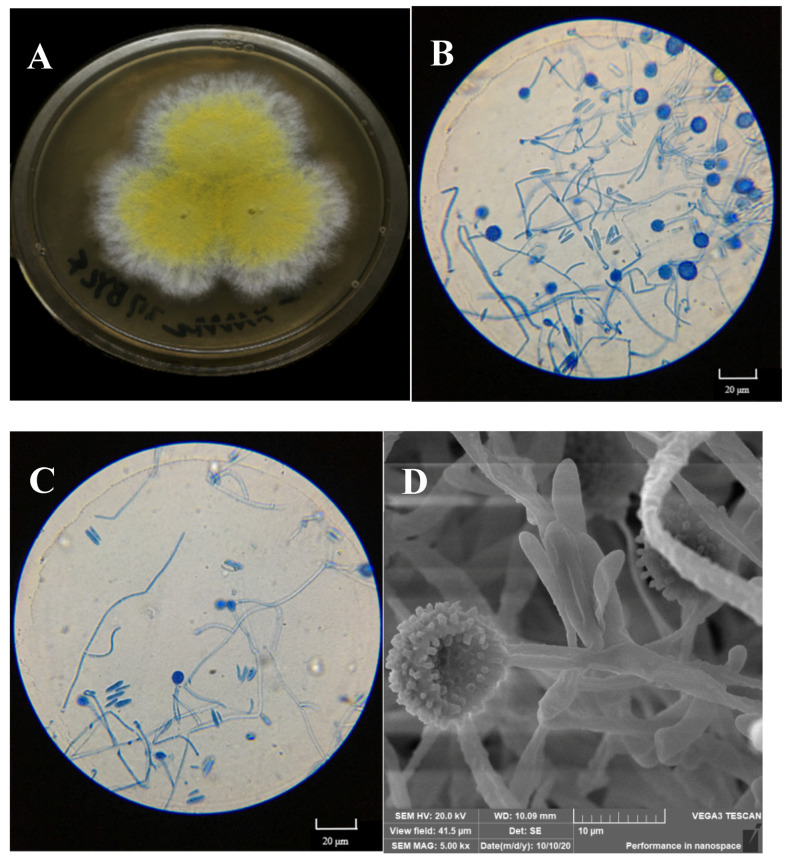
Macroscopic features of the isolated mycoparasitic fungus, *Hypomyces chrysospermus*. (**A**) Colonial morphology on PDA, (**B**,**C**): Optical microscopy images of the morphology of mycelia and conidiophore and chlamydospores; (**D**) scanning electron micrographs of mycelia and chlamydospores).

Up to now, few studies about the *B. griseus*–mycoparasitic fungus association have been carried out. The symptoms of mycoparasitic fungus infection of *B. griseus* in this study differed from those of other bolete mushrooms, as studies found that infections of the genus *Hypomyces* led to total necrosis of bolete hosts [18]. Considering the strong Cd bioaccumulation capacity of *B. griseus*, even in natural habitats without Cd contamination, the unique biological strategy of *B. griseus* to take up Cd from the soil matrix was assumed, such as being parasitized by fungicolous fungi and forming a symbiote. Thus, this study aimed (1) to test and analyze the Cd contents in normal-developed sporocarps and symbiotic fruiting bodies of *B. griseus*–mycoparasitc fungus, (2) to isolate and identify the mycoparasitc fungus from normal-developed sporocarps (Figure 1) and symbiotic fruiting bodies of *B. griseus*–mycoparasitc fungus (Figure 2), and (3) to assess the Cd tolerance and Cd-accumulating traits of isolated mycoparasitic fungus. This study could help to exploit the possible mechanism of Cd bioaccumulation of *B. griseus*, from the perspective of the roles of the fungi-associated fungi in Cd uptake.

## 2. Material and Methods

### 2.1. Sampling

Samples of normal-developed sporocarps and symbiotic fruiting bodies of *B. griseus*–mycoparasitic fungus (marked as deformed sporocarps) were collected from four locations separated by approximately 20–40 km, being Guishan Town, Shilin Town, Banqiao Town, and Changhu Town in Shilin City, Yunnan Province, China, in June and July of 2020 and 2021. Five to six areas that were at a distance from the communication routes and other sources of environmental pollution and rich in *B. griseus* were selected from each location. During the sampling, attention was paid to make sure that the normal-developed and deformed sporocarps were collected from the same area (within approximately 10 ha^−1^) and at the same time (a single day). In total, 22 samples of normal-developed sporocarps and 22 samples of deformed sporocarps were collected from 22 studied areas. From each of the studied areas, 10–15 fruiting bodies were collected and pooled for each sample.

The samples were brought to Kunming University of Science and Technology, Wild Edible Mushroom Research Laboratory, where the samples were photographed. Then, three normal-developed sporocarps and one deformed sporocarp were randomly selected for the isolation and identification of mycoparasitic fungus. The rest of the sporocarps were cleaned to remove forest debris and washed successively with running water and distilled water. The cleaned sporocarps were freeze dried and ground into a fine powder (40 meshes) and stored in sealed polyethylene bugs in a vacuum dryer for Cd determination.

### 2.2. Cd Determination

The Cd contents of normal-developed and deformed sporocarps were determined by atomic absorption spectrometry [2]. Briefly, 0.1 g samples were soaked overnight in open polytetrafluoroethylene (PTFE) vessels with 8 mL of concentrated nitric acid (65%). Then, after pre-digestion was conducted using a digestion apparatus at 120 °C for 60 min, the PTFE vessels were closed and heated in a microwave oven (MARS6, CEM Corp., Charlotte, NC, USA). The digestion conditions were set at 1.6 kW power by a 3-step heating program, being 8 min ramp, temperature of 120 °C, and 5 min hold; 5 min of ramp, temperature of 150 °C, and 5 min hold; 8 min ramp, temperature of 190 °C, and 25 min hold. The digest was diluted and filtered on 0.45 μm polyethersulfone membrane filters. Then, the solution was determined by an atomic absorption spectrophotometer (AAS400G, Analytik Jena AG, Jena, Germany) with a deuterium background corrector. The analytical wavelengths were determined at 228.8 nm. The pyrolysis and atomization temperatures were 350 and 1300 °C, respectively. Signals were measured as the peak area. The assurance quality of the analytical method was investigated through analysis of the certified reference materials of GBW10025 (CRM spirulina) supplied by Geophysiochemistry Prospecting Institute of Academy of Geological Science of China. The certified value for Cd of CRM spirulina was 0.37 mg·kg^−1^. CRM was analyzed for Cd with each analytical batch of samples in triplicate, and the recovery of Cd was 92.5–106.04%. Three replicates were conducted for the Cd determination of the samples.

### 2.3. Isolation of Mycoparasitic Fungus

All selected normal and deformed sporocarps from Section 2.1 were cleaned to remove forest debris, washed with running water to remove dirt, subjected to surface sterilization sequentially with 75% ethanol for 90 s and 5% NaCl 3 times, and then rinsed in sterile water 3 times.

For normal sporocarps, small pieces (0.5 cm × 0.5 cm × 0.5 cm) from under the epidermis were taken from the base of the stipes. The pieces were placed on potato dextrose agar (PDA) plates and incubated at 28 °C in the dark for 5 days.

For deformed sporocarps, a flame-sterilized loop was used to pick out spores (Figure 2) on PDA plates. The plates were incubated at 28 °C in the dark for 5 days.

After 5 days, the leading edges of the fungal colonies was transferred to PDA plates and incubated at 28 °C in the dark for 5 days for growth and isolation.

### 2.4. Morphological Identification and Taxonomic Analyses of Mycoparasitic Fungus

The morphological characteristics were recorded, including the colony shape, size, color, surface state, and edge. The microstructures of the endophyte mycelium and spores were examined using a light microscope after being stained bright blue with gossypol blue dye.

The genomic DNA of the isolated fungus was extracted using a Trelief Plant Genomic DNA Kit (Tsingke Biotechnology Co., Ltd., Beijing, China). The ITS1-ITS4 region was amplified using PCR primers ITS1 (5′-TCCGTAGGTGAACCTGCGG-3′) and ITS4 (5′-TCCTCCGCTTATTGATATGC-3′) [19]. Electrophoresis was carried out using agarose gel. The PCR products were purified using PCR purification kits (Tsingke Biotechnology Co., Ltd., Beijing, China) and sequenced using a BigDye Terminator v3.1 Cycle Sequencing Kit (Applied Biosystems, Waltham, MA, USA). Raw sequences were aligned using ContigExpress (Vector NTI Suite8.0; Invitrogen) and BLAST searched for the best match in NCBI (blast.ncbi.nlm.nih.gov accessed on 2 March 2019).

### 2.5. Determination of the Cd Resistance of Mycoparasitic Fungus

The Cd resistance of the isolated mycoparasitic fungus was examined using the PDA plates with gradually increasing concentrations of CdCl_2_, with Cd^2+^ of 0 (control), 80, 120, 160, and 400 mg·L^−1^. Six replicates were conducted for each Cd treatment. The plates were incubated at 28 °C in the dark for 5 days. The concentration of Cd^2+^ at which no visible fungal growth was observed was considered the minimum inhibitory concentration (MIC) of the mycoparasitic fungus. The Cd^2+^ level just below the MIC was considered as the highest Cd^2+^ concentration tolerated by the mycoparasitic fungus. Then, the inhibition percentage (IP, %) of Cd^2+^ of the fungus was measured by the ratio of the colony diameter with Cd^2+^ treatment to the colony diameter of the control [20]. EC_50_ refers to the Cd^2+^ concentration required to inhibit 50% of the fungal growth [21].

### 2.6. Effects of Cd^2+^ on the Morphology of Mycoparasitic Fungus

Scanning electron microscopy (SEM) was employed to investigate the micromorphology of the mycelia from the colonies of the 0–160 mg·L^−1^ Cd^2+^ treatments [22]. Briefly, the colonies of the mycoparasitic fungus from PDA plates with 0–160 mg·L^−1^ Cd^2+^ were collected, and the mycelia was fixed and used as the samples. Images of the mycelia under different Cd^2+^ concentrations were obtained via SEM analysis (VEGA3-SBH, Tescan) under the following analytical conditions: HW = 25.0 kW, WD = 14.77 mm, and SignalA = SE.

### 2.7. Effects of Cd^2+^ on the Growth of Mycoparasitic Fungus

In accordance with the highest Cd^2+^ concentration tolerated by the fungus, the effects of Cd^2+^ on the growth of the fungus were examined using shake flasks with 100 mL potato dextrose broth (PDB) added with gradually increased concentrations of CdCl_2_, with Cd^2+^ of 0 (control), 20, 40, 60, 80, 100, 120, 140, and 160 mg·L^−1^. Six replicates were conducted for each Cd^2+^ treatment. Cultures were incubated at 28 °C and 120 rpm for 7 days. Then, the pH values of the fermentation mixtures were determined using a pH meter. The mixture was filtered, and the mycelia were collected and washed three times with deionized water. The fresh mycelia from the three fermentation mixtures were freeze dried. The dried mycelia were accurately weighed to determine the effects of Cd^2+^ on the biomass yields. The fresh mycelia from the rest of the three fermentation mixtures was pooled for the investigation of the contents of soluble protein and the activities of antioxidant enzymes, such as superoxide dismutase (SOD), peroxidase (POD), and catalase (CAT) [22].

### 2.8. Cd^2+^ Absorption by Mycoparasitic Fungus

The dried mycelia from Section 2.7 were digested, and the Cd contents in the mycelia were determined, as described in Section 2.2. The values of Cd^2+^ absorption by the mycoparasitic fungus during the incubation were calculated as mg Cd per g dried mycelia.

### 2.9. Data Analysis

Data were analyzed by SPSS 16.0 (SPSS Inc., Chicago, IL, USA). The differences between means were analyzed using Duncan’s multiple range test or Student’s t-test at the 0.05 probability level. Graphical work was conducted on Excel 2007 (Microsoft, Redmond, WA, USA). The results were expressed as mean ± SD.

## 3. Results and Discussion

### 3.1. Cd Contents in Normal and Deformed Sporocarps of B. griseus

As shown in Table 1, the Cd contents in 22 normal-developed sporocarps of *B. griseus* ranged from 13.71–43.40 mg·kg^−1^, with an average of 24.45 mg·kg^−1^. According to a previous study [2], the Cd contents in 227 *Boletaceae* mushrooms from Yunnan Province ranged from not detected in *B. aereus* to 25.01 mg·kg^−1^ DW in *B. griseus*. The results of the present paper are consistent with those of the previous study [2]. Researchers have reported the contents of Cd in *Boletaceae* mushrooms, such as 1.2 mg·kg^−1^ in *B. edulis* [23]; 2.77 mg·kg^−1^ in *B. edulis* [13]; 0.58–1.31 mg·kg^−1^ in different parts of *B. aereus*, *B. aestivalis*, *B. edulis*, and *B. pinophilius* [24]; 1.44–2.01 mg·kg^−1^ in different parts of *B. badius* [25]; 0.24 mg·kg^−1^ in *B. aereus* [26]; and 0.28 mg·kg^−1^ in *Neoboletus erythropus* [27].

The Cd contents in 22 deformed sporocarps of *B. griseus* ranged from 6.35–49.29 mg·kg^−1^, with an average of 26.75 mg·kg^−1^. All the normal-developed and deformed sporocarps of *B. griseus* significantly accumulated high amounts of Cd. According to Student’s t-test, among the 22 samples, 13 deformed sporocarps had significantly higher Cd contents than the normal-developed sporocarps.

In the last decade, extensive studies have shown that symbiotic association, such as mycorrhizas of fungi and plants, could enhance the tolerance and accumulation abilities of hosts regarding heavy metals (HMs) [28]. Mutualistic symbiotic fungi, such as ectomycorrhizal fungi (EMF), arbuscular mycorrhizal fungi (AMF), dark septate endophytes (DSEs), and endophytic fungi, are considered to be the remarkable factors of metal-accumulating plant species [20,29,30,31,32]. Co-culturing of fungi–fungi was recently established to produce new secondary metabolites. Wang et al. reported that mycelial pellets (*Aspergillus fumigatus*) and *Synechocystis* sp. PCC6803 comprised a fungus–microalgae symbiotic system, assisting microalgae flocculation and immobilization and the adsorption behavior for HMs [33]. The co-cultivation of microalgae with filamentous fungi is a superior method to efficiently accumulate and harvest the total biomass, and to remove pollutants from water [34]. However, the interactions of fungi associated with fungi have not yet been fully investigated, except for morphological and anatomical studies and molecular phylogenetic analyses of mycoparasitic fungi [35].

### 3.2. Isolation and Identification of Mycoparasitic Fungus

Fungal outgrowths from the surface-sterilized tissues of normal sporocarps and the spores of deformed sporocarps were observed after incubation on PDA plates. Only one filamentous fungal colony was isolated on the basis of unique phenotypic characteristics, as shown in Figure 3A.

The colony on PDA medium was white, and it had a flat appearance with a regular edge. Then, the mycelia differentiated into yellow spores after approximately 3 days. As shown in Figure 3B,C, the mycelium was thin-walled and hyaline. Conidia were observed to be 10–20 × 3–8 μm in size, elliptical, aseptate, and single-celled, with smooth and thin walls, and hyaline. Globular tissues were observed to be approximately 10 μm in diameter under optical microscopy. SEM showed that the globular tissues were chlamydospores with thick walls and were prominently verrucose (Figure 3D).

According to the sequence of the ITS1-5.8S-ITS4 region, the isolated fungus belonged to the Ascomycota (classes: *Sordariomycetes*, *Hypocreales*, *Hypocreaceae*, and *Hypomyces*), and it was identified as *Hypomyces chrysospermus* based on a blast analysis in the NCBI GenBank database (MK560123.1).

*H. chrysospermus* is a cosmopolitan parasite of many boletes. According to Kaygusuz et al. [18], *Hypomyces* is easy to identify as the species in this genus usually produce bright-colored perithecia, conidiophores, and chlamydospores, and infection of *H. chrysospermus* causes decay of the host. However, *H. chrysospermus* seems to play an essential role in the development of sporocarps of *B. griseus*. *H. chrysospermus*-infected or uninfected *B. griseus* could develop normal fruiting bodies that had a distinguished gray color and were cap- and stipe-shaped. Meanwhile, under certain environmental conditions (temperature and relative humidity), deformed fruiting bodies with a significantly bigger size and weight usually formed, being a symbiont of *B. griseus*–*H. chrysospermus*. The symbiont had no shape features of bolete mushrooms, and it was usually spherical with a gray-white appearance, as shown in Figure 2.

### 3.3. Cd Tolerance of H. chrysospermus

**Figure 4:**
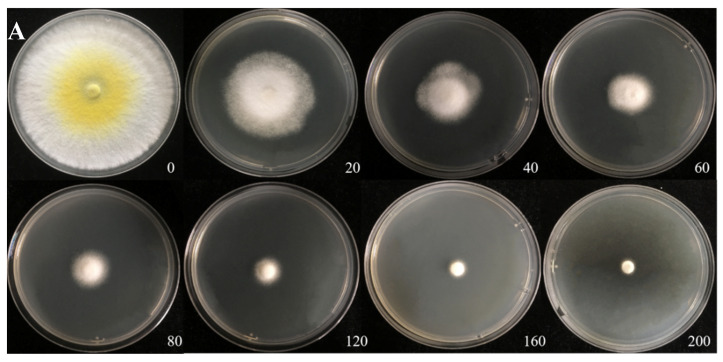

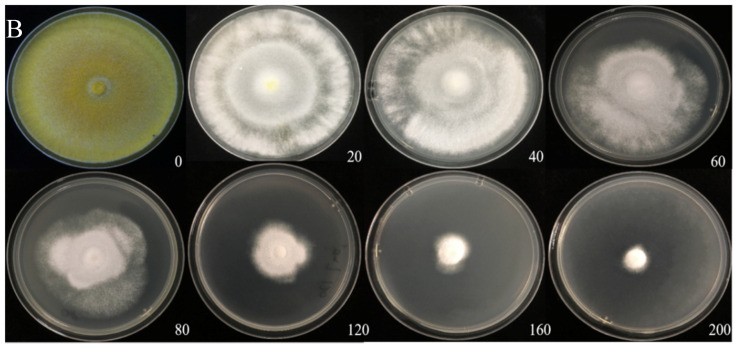
Cadmium tolerance of *H. chrysospermus* on PDA plates. (**A**) 5 days; (**B**) 10 days. Data in the figure indicate Cd^2+^ concentrations (mg·L^−1^) in PDA.

**Figure 5:**
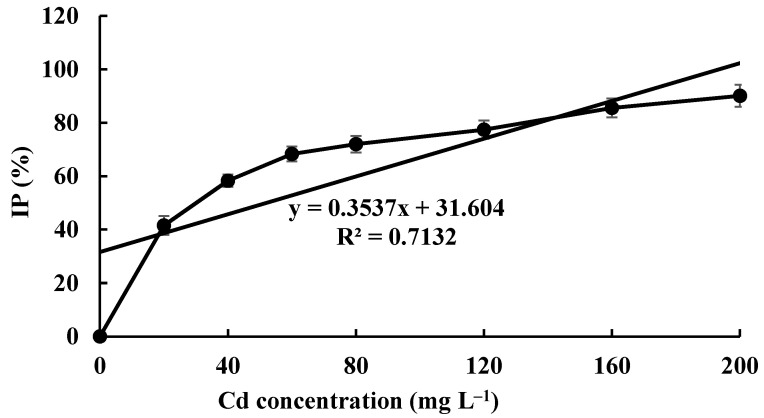
The inhibition percentage of Cd^2+^ at different concentrations on *H. chrysospermus*.

The Cd tolerance of *H. chrysospermus* was determined on Cd-enriched PDA plates. As shown in Figure 4, *H. chrysospermus* grew very rapidly when cultured on PDA. When cultured for 5 days on PDA without Cd^2+^, the diameter of the colonies was 7 cm on average (Figure 4A). Cd significantly inhibited *H. chrysospermus.* Colonies showed an inverse relationship with the Cd^2+^ concentrations, and the inhibition percentage showed a direct positive correlation with the Cd^2+^ concentrations, with R^2^ being 0.7132. The highest Cd^2+^ concentration tolerated by *H. chrysospermus* was 160 mg·L^−1^. The MIC value of Cd^2+^ to *H. chrysospermus* was determined to be 200 mg·L^−1^, and the EC_50_ was calculated to be 52 mg·L^−1^, according to the IP of Cd to *H. chrysospermus* (Figure 5). However, when cultured for a prolonged time, growing mycelium could be observed on the PDA of 200 mg·L^−1^, as shown in Figure 4B. Traxler et al. described a similar phenomenon; that is, the growth of *Schizophyllum commune* was still visible even at the highest metal concentrations used in PDA medium cultivated for 14 days [36].

**Figure 6:**
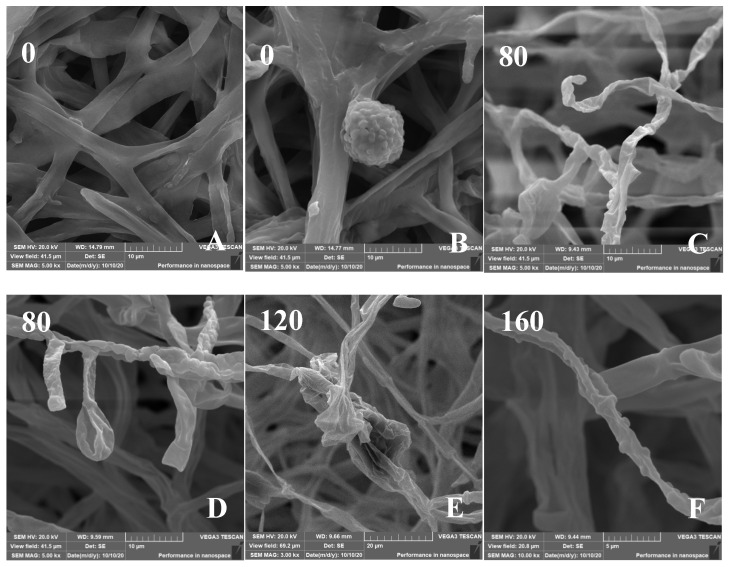
(**A**–**F**) Scanning electron micrographs of *H. chrysospermums* grown for 7 days on PDA with gradually increasing concentrations of Cd^2+^. Data of 0–160 in the figures indicate the Cd^2+^ concentrations in PDA (mg·L^−1^).

The effect of Cd on the micromorphology of the mycelia was investigated (Figure 6). The SEM images of control *H. chrysospermus* showed long and rope-like fungal hyphae, which were highly branched and intertwined with one another, and no physical damage was observed (Figure 6A). Meanwhile, fully developed chlamydospores with thick walls and prominent verrucose features were observed, as shown in Figure 3D and Figure 6B. The presence of Cd resulted in locally twisted (Figure 6C) and deformed chlamydospores (Figure 6D). The increased Cd treatment distorted and shrunk the cell walls of the fungal mycelium (Figure 6E,F). The mycelia were partly swollen when treated with 160 mg·L^−1^ of Cd (Figure 6F). This finding may be due to the immobilization effect of the cell wall via Cd^2+^ binding by functional groups, such as carboxyl (-COOH), hydroxyl (-OH), carbonyl (-COH), and amino (-NH_2_), to form precipitation on the cell surface [37,38]. Studies have shown that HMs have various effects on the composition of microbial cell components. The uptake and accumulation of HMs often causes significant damage at the morphological, cellular, physiological, and molecular levels. Cd is one of the most toxic metals among HMs. Sharma et al. reported that the fungal mycelium was slightly broken with some visual deformities [39]. However, the shape was distinguishable and well-regulated in the presence of Pb and Ni, whereas the presence of Cd distorted and shrunk the cell walls of *Phlebia brevispora*.

Deng et al. characterized the features of a Cd-, Pb-, and Zn-resistant endophytic fungus *Lasiodiplodia* sp. MXSF31 from metal-accumulating *Portulaca oleracea*, and the strain was resistant to 5 mM Cd [40]. Mohammadian et al. studied different filamentous fungi isolated from contaminated mining soil and showed different profiles [41]. Among them, *Trichoderma harzianum* showed the maximum MIC value for Cd, being 35 mg·L^−1^. Albert et al. evaluated the tolerance of the soil fungus *Absidia cylindrospora* against three trace metals, namely, Cd, Cu, and Pb, before considering a possible use to treat contaminated soils. The concentration that inhibited 50% of fungal growth (IC_50_) was 100 mg·L^−1^ for Cd [21].

Cd is toxic to cells, and organisms show visible toxicity symptoms under Cd stress higher than 5 mg·kg^−1^. However, mushrooms are characterized by a high trace element accumulation capacity, especially in the case of Cd, Pb, Cu, and Zn [24,42]. Numerous papers showed that the contents of many trace elements, especially Cd and Hg, increased in mushrooms from polluted areas compared with those from unpolluted rural sites [24]. Some plant species, such as *Solanum nigrum*, *Solanum photeinocarpum*, and *Siegesbeckia orientalis*, were studied as Cd hyperaccumulators and accumulators, which could be used for phytoremediation of Cd-contaminated soil. These plant species were usually found in different ecotypes with quite different morphology and Cd accumulation, including the mining ecotype (accumulating ecotype) and the farmland ecotype (non-accumulating ecotype) [43,44]. However, *B. griseus* showed a ubiquitous Cd-accumulating capacity, which seemed unaffected by the natural habitat. Considering the Cd tolerance of *H. chrysospermus* isolated from normal and deformed sporocarps of *B. griseus*, the Cd absorption of *H. chrysospermus* could be assumed.

### 3.4. Cd Absorption by H. chrysospermus

**Figure 7:**
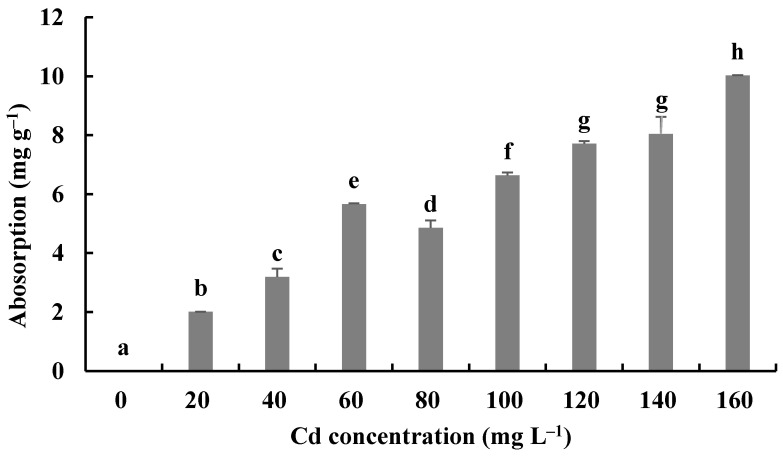
Cd^2+^ absorption by *H. chrysospermus.* Different letters indicated significant difference (*p* < 0.05).

The Cd bioaccumulation of *H. chrysospermus* was assessed in relation to the initial Cd^2+^ concentrations (Figure 7). The Cd contents in dried biomass of *H. chrysospermus* increased with rising Cd^2+^ concentrations in PDB medium. The maximum uptake value was 10.03 mg·g^−1^. Li et al. described a similar phenomenon and reported that the Cd concentrations that accumulated in the hyphae of *Pleurotus ostreatus* HAU-2 increased with increasing concentrations of Cd in the liquid culture [22].

In comparison, the maximum values of Cd uptake by fungi in the liquid culture have been reported to be 4.6 × 10^4^ mg·kg^−1^ for *Lasiodiplodia* sp. MXSF31 [40], 1.89 mg·g^−1^ for *P. ostreatus* [22], 9 μg·mg^−1^ for *A. cylindrospora* [21], and 2.3−11.9 mg·g^−1^ for 5 fungal strains, with the highest Cd tolerance shown by soybean and barley [45]. During the experiment described here, *H. chrysospermus* isolated from *B. griseus* showed a significant absorption capacity for Cd^2+^, and it may be a promising Cd bioabsorbent for bioremediation.

### 3.5. Effects of Cd on the Growth of H. chrysospermus

**Figure 8:**
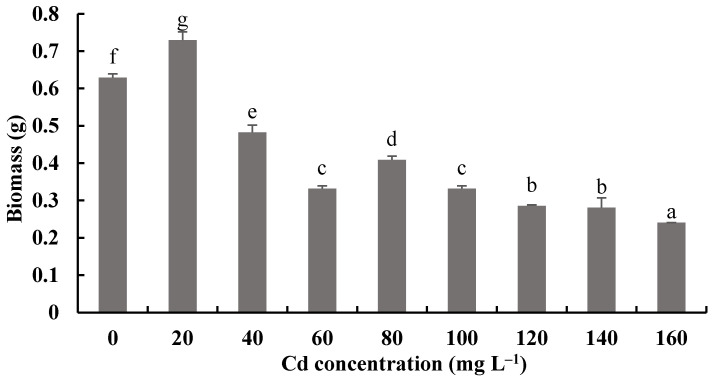
Effects of different concentrations of Cd^2+^ on the biomass of *H. chrysospermus.* Different letters indicated significant difference (*p* < 0.05).

**Figure 9:**
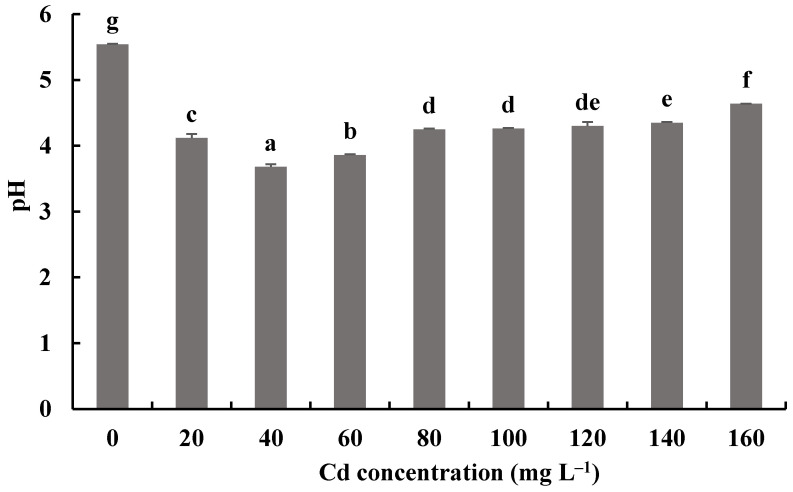
Effects of different concentrations of Cd^2+^ on the pH of a fermentation mixture of *H. chrysospermus.* Different letters indicated significant difference (*p* < 0.05).

Figure 8 and Figure 9 show the biomass production and pH measurements of *H. chrysospermus* cultured for 7 days in PDB with different Cd^2+^ concentrations. When the Cd^2+^ concentration in the PDB medium increased from 0 to 20 mg·L^−1^, the biomass of *H. chrysospermus* increased significantly, being 0.73 mg DW. Then, the biomass decreased with increased Cd^2+^ concentrations up to 160 mg·L^−1^. Li et al. revealed that low-concentration Cd (<20 mg·L^−1^) and Cr (<150 mg·L^−1^) did not notably suppress the hypha growth of *P. ostreatus* HAU-2, but higher concentrations of Cd and Cr evidently suppressed it [22]. Sharma et al. demonstrated that an enhancement in the fungal biomass of *P. brevispora* was observed with an increased metal concentration up to 1, 4, and 10 μmol·L^−1^ for Cd, Ni, and Pb, respectively, which then declined with a further increase in the metal concentrations [39].

The pH values of fermentation mixtures of *H. chrysospermus* with and without Cd^2+^ all decreased compared with the initial pH of 6.19. Meanwhile, the pH values of fermentation mixtures with Cd^2+^ were significantly lower than those of the control. The lowest pH value of 3.68 was shown by the fermentation mixtures with 40 mg·L^−1^ Cd^2+^, a decrease by 2.51. Fungi can secrete inorganic acid and organic acid compounds to alleviate the toxic effects of HMs [46]. Wang et al. showed that *P. ostreatus* ISS-1 secreted oxalic acid, citric acid, and formic acid regardless of Pb exposure, but the oxalic acid content was significantly higher under Pb stress than that in the control [47]. Organic acids (citric acid and oxalic acid) chelate with toxic metal ions and form precipitation.

**Figure 10:**
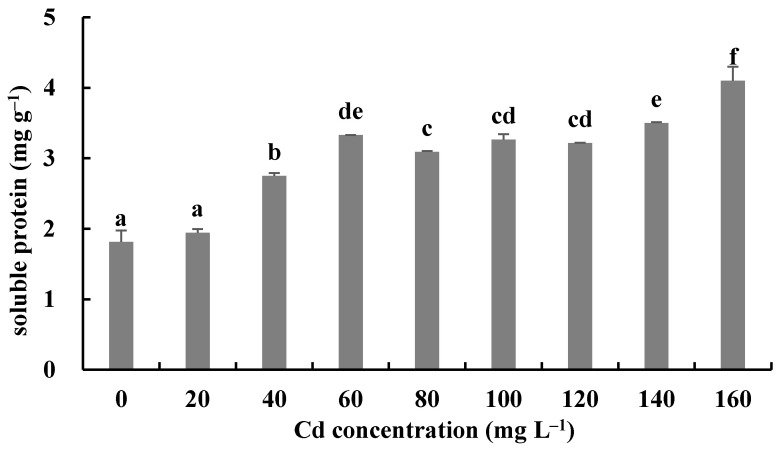
Effects of different concentrations of Cd^2+^ on soluble protein of *H. chrysospermus.* Different letters indicated significant difference (*p* < 0.05).

**Figure 11:**
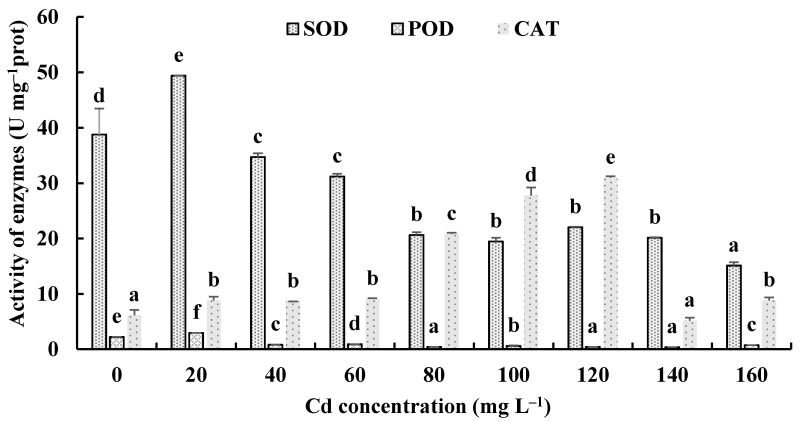
Effects of different concentrations of Cd^2+^ on the activities of antioxidant enzymes of *H. chrysospermus*. Different letters indicated significant difference (*p* < 0.05).

The responses of the soluble protein and antioxidant enzymes of the fungus with different Cd^2+^ concentrations were assayed to further investigate the tolerance factors of *H. chrysospermus* to Cd^2+^. As shown in Figure 10, the soluble protein contents of *H. chrysospermus* significantly increased with increased Cd^2+^ concentrations. Figure 11 shows the effects of different concentrations of Cd^2+^ on the activities of the antioxidant enzymes of *H. chrysospermus*. The SOD activity was sensitive to Cd^2+^ treatment, showing a significant increasing tendency for 20 mg·L^−1^ Cd^2+^, but it decreased with the increasing Cd^2+^ concentration. *H. chrysospermus* in this study showed a significantly lower POD activity [22], which was suppressed by Cd^2+^ treatments. The CAT activities under Cd stress were higher than those in the control group. The CAT activity reached a maximum value of 30.97 U·mg^−1^ prot with a Cd^2+^ concentration of 120 mg·L^−1^. The exposure of fungal species to HMs induces stress conditions, resulting in the production of reaction oxygen species, such as superoxide, peroxides, and hydroxyl radicals, that damage fungal cell and organelle structures and alter metabolism [22,46]. Thus, the fungi of HM-resistant species need to adopt strategies to resist oxidative stress. The cellular immune system is the basic strategy employed to resist metal toxicity. Oxidized enzyme species, such as SOD, POD, and CAT, are important components of the cellular immune system, and they exert a significant effect on the removal of cellular active oxygen. Li et al. revealed that the concentrations of these three enzymes first increased and then decreased in the presence of Cd and Cr, indicating that oxidized enzyme species could be induced by relatively low concentrations of Cd or Cr, thus playing a role in the removal of active oxygen [22]. However, overly high concentrations of HMs may severely damage cells, suppressing enzymes.

## 4. Conclusions

To the authors’ best knowledge, this work is the first study to identify the symbiotic association of *B. griseus* and *H. chrysospermus* and elucidate the Cd-resistant characteristics of the isolated strain of *H. chrysospermus* from *B. griseus*. This study further confirms the Cd bioaccumulation capacity of *B. griseus*. The isolated strain of *H. chrysospermus* from *B. griseus* showed a strong ability to tolerate Cd, and the maximum Cd uptake reached 10.03 mg·g^−1^. In general, the time required for fruiting body differentiation, including induction, development, and maturation, in mushroom-forming fungi is about 4–12 days. Considering the strong Cd bioaccumulation capacity of *B. griseus*, even in natural habitats without Cd contamination, the symbiotic association of *H. chrysospermus* might represent the biological strategy adopted by *B. griseus* to promptly and efficiently take up Cd from the soil matrix. The MIC of Cd of the isolated strain of *H. chrysospermus* was 200 mg·L^−1^. However, the growth of the strain was still visible after prolonged culturing. The immobilization effects of the cell wall and acid compounds and antioxidant enzymes were employed by the fungi to alleviate the toxic effects of Cd. Thus, this study not only provides new insight into the Cd bioconcentration mechanisms of *B. griseus* but also provides a potential bioremediation fungus for Cd contamination.

## Figures and Tables

**Table 1:** Cadmium contents of 22 samples of normal and deformed sporocarps of *B. griseus* (mg·kg^−1^ DW).

Sample ID	Normal	Deformed	Sample ID	Normal	Deformed
1	26.1 ± 0.0 ^a^	14.9 ± 0.0 ^b^	13	16.1 ± 0.1 ^b^	35.4 ± 0.0 ^a^
2	21.1 ± 4.9 ^a^	13.5 ± 0.8 ^b^	14	21.3 ± 0.0 ^a^	16.2 ± 0.1 ^b^
3	18.8 ± 1.4 ^b^	25.3 ± 0.5 ^a^	15	43.4 ± 0.0 ^b^	44.4 ± 0.1 ^a^
4	27.7 ± 1.7 ^a^	25.6 ± 1.6 ^a^	16	27.5 ± 0.2 ^b^	49.3 ± 0.9 ^a^
5	15.6 ± 0.1 ^b^	27.6 ± 1.0 ^a^	17	19.3 ± 0.0 ^b^	19.6 ± 0.0 ^a^
6	33.4 ± 0.8 ^b^	40.3 ± 0.0 ^a^	18	27.2 ± 0.1 ^b^	36.5 ± 0.6 ^a^
7	24.7 ± 0.6 ^a^	6.4 ± 0.2 ^b^	19	21.3 ± 0.1 ^b^	24.1 ± 0.1 ^a^
8	15.2 ± 0.0 ^a^	8.4 ± 0.6 ^b^	20	17.8 ± 0.2 ^b^	33.4 ± 0.1 ^b^
9	37.2 ± 1.3 ^a^	30.2 ± 2.8 ^b^	21	13.7 ± 0.0 ^a^	10.8 ± 0.1 ^b^
10	31.6 ± 2.9 ^b^	45.8 ± 0.3 ^a^	22	21.4 ± 0.3 ^a^	21.3 ± 0.2 ^a^
11	35.3 ± 0.5 ^a^	17.9 ± 0.1 ^b^	Average	24.5 ± 7.9 ^a^	26.3 ± 12.4 ^a^
12	22.1 ± 0.0 ^b^	31.7 ± 0.3 ^a^			

Footnote of ^a^ and ^b^ in each line indicate significant differences (*p* < 0.05).
